# Improving the intake of nutritious food in children aged 6-23 months in Wuyi County, China – a multi-method approach

**DOI:** 10.3325/cmj.2013.54.157

**Published:** 2013-04

**Authors:** Qiong Wu, Michelle H.M.M.T. van Velthoven, Li Chen, Josip Car, Diana Rudan, Vanja Saftić, Yanfeng Zhang, Ye Li, Robert W. Scherpbier

**Affiliations:** 1 Department of Integrated Early Childhood Development, Capital Institute of Pediatrics, Beijing, China; 2 Global eHealth Unit, Department of Primary Care and Public Health, Imperial College London, London, United Kingdom; 3 University Hospital Dubrava, Zagreb, and Faculty of Medicine, University of Split, Split, Croatia; 4 University Hospital “Sisters of Mercy,” Zagreb, Croatia; 5 Section of Health and Nutrition, Water, Environment and Sanitation, UNICEF China, Beijing, China

## Abstract

**Aim:**

To develop affordable, appropriate, and nutritious recipes based on local food resources and dietary practices that have the potential to improve infant feeding practices.

**Methods:**

We carried out a mixed methods study following the World Health Organization’s evaluation guidelines on the promotion of child feeding. We recruited caregivers with children aged 6-23 months in Wuyi County, Hebei Province, China. The study included a 24-hour dietary recall survey, local food market survey, and development of a key local food list, food combinations, and recipes. Mothers tested selected recipes at their homes for two weeks. We interviewed mothers to obtain their perceptions on the recipes.

**Results:**

The 24-hour dietary recall survey included 110 mothers. Dietary diversity was poor; approximately 10% of children consumed meat and only 2% consumed vitamin A-rich vegetables. The main reason for not giving meat was the mothers’ belief that their children could not chew and digest meat. With the help of mothers, we developed six improved nutritious recipes with locally available and affordable foods. Overall, mothers liked the recipes and were willing to continue using them.

**Conclusions:**

This is the first study using a systematic evidence-based method to develop infant complementary recipes that can address complementary feeding problems in China. We developed recipes based on local foods and preparation practices and identified the barriers that mothers faced toward feeding their children with nutritious food. To improve nutrition practices, it is important to both give mothers correct feeding knowledge and assist them in cooking nutritious foods for their children based on locally available products. Further research is needed to assess long-term effects of those recipes on the nutritional status of children.

Undernutrition and a poor nutritional status of infants and young children is highly prevalent in low- and middle- income countries and results in substantial mortality and morbidity (1). Although China has made huge improvements in child nutrition, still many children have a poor nutritional status, which, except in the poorest counties, is not related to food insecurity (2). In China, the prevalence of stunted infants aged 6-12 months was estimated to be 12.5% and the prevalence of anemia in children under age 24 months was between 30% and 40% in poorer rural areas in 2009 (3). Moreover, the prevalence of stunted and anemic children was higher in poor rural areas than in rural areas with average incomes (3,4).

Appropriate child feeding is the basis for a good nutritional status and healthy development, and a key factor for health in later life (5,6). The critical window for child nutrition is from pregnancy through the first 24 months of life; any deficits during this time can cause irreversible damage (1,7). Undernutrition is a significant risk factor for illnesses such as infections (1). Therefore, the World Health Organization (WHO) and United Nations Children’s Fund (UNICEF) recommend that infants should be exclusively breastfed from birth to six months and be continuously breastfed till they are two years old or above (8). Also, infants should be given complementary food from six months of age (8).

During the past 10 years, China has adopted the WHO’s feeding recommendations and implemented programs to improve nutrition practices, such as the infant and young child feeding guidelines (9) and the integrated management of childhood illness guidelines (10). Nevertheless, in rural China many infants and young children do not receive adequate breastfeeding and complementary feeding. The proportions of children who were exclusively breastfed for the first 6 months of life, introduced to complementary foods at 6-9 months, and continuously breastfed during 12-15 months were only 27.6%, 43.3%, and 37.0% respectively (11). In addition, a too early and too late introduction of complementary foods (12-16) and a restriction in foods selection such as animal source foods (14-16) are still widespread in China. Studies show that children in rural China are often fed food that mainly contains carbohydrates and lacks in protein and fat (17). Caregivers do not give available quality foods such as meat, vegetables, oil, and eggs to their children due to traditional beliefs that “baby is too small and cannot digest it” (18). More efforts are required to improve the nutritional status of children and as part of this, it is necessary to improve child feeding practices (19,20). The poor complementary feeding practices indicate that the general recommendations in the infant feeding guidelines have not been put into practice. However, how to translate general recommendations into a specific context in China has rarely been explored. In this article, we aimed to develop affordable, appropriate, and nutritious recipes based on local food resources and dietary practices. We intended to provide guidance and support to mothers to feed their children with nutritious foods. This work can inform others to develop specific interventions that may have potential to improve the nutritional status of young children.

## Methods

### Study design

**Figure 1:**
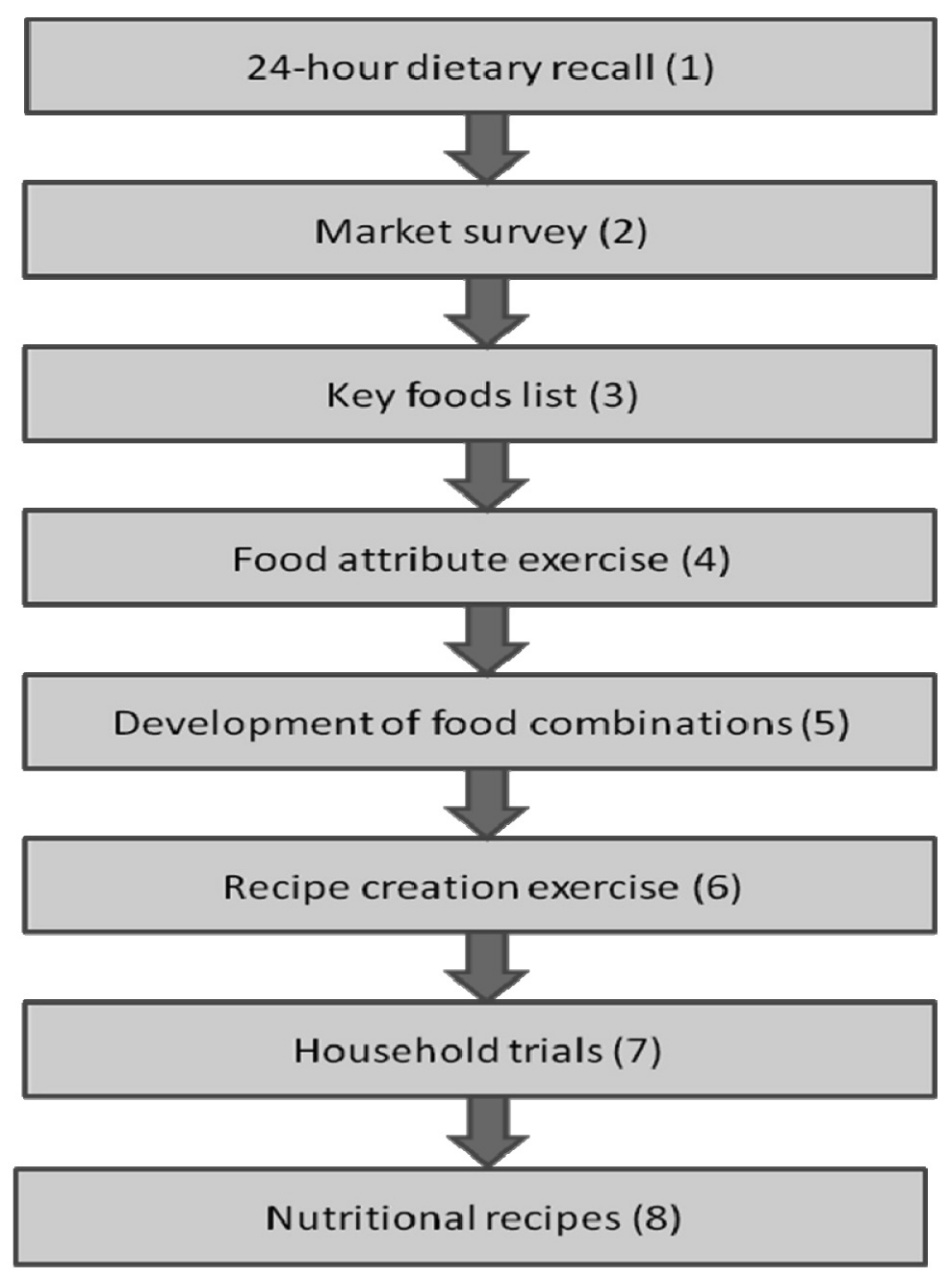
Flowchart for development of nutritional recipes.

We used multiple (both quantitative and qualitative) methods following the WHO guidelines on the promotion of child feeding (21). The study was structured in eight parts (Figure 1): 1) We did a 24-hour dietary recall survey, which provided us with information on feeding practices, and a list of foods that the local children frequently consumed; 2) We did a market survey in five different local markets where mothers usually bought their foods; 3) We obtained the prices and information on the seasonality of those foods and developed a key food list; 4) We gained a better understanding of the mothers’ perceptions on the key foods and how they cooked these foods; 5) We created nutritious food combinations for children; 6) We invited mothers to cook the combinations using their own cooking style and asked them and their children to taste the foods and give comments; 7) We requested mothers to test ten recipes to assess the acceptability, feasibility, and compliance (the number of times they used them) of these recipes; 8) We selected six improved recipes and developed a recipe booklet for local children aged 6 to 23 months.

### Study setting

The study took place in Wuyi County, Hebei province from July till December 2011. Hebei province is located in the northern part of North China and Wuyi County is located in the central southern part of the Province. In 2010, the county had a total population of 310 000 and under-five population of 18 000 (unpublished data). Wuyi County is one of the poverty-stricken counties in China (22). The annual per capita net income of rural residents was 3039 CNY (482 USD), which is much lower than the national average of 5919 CNY (939 USD) (23).

### Participants and recruitment

There were two types of participants in this study: 1) mothers with children aged 6 to 23 months; 2) children aged 6 to 23 months. Local coordinators helped us to find the mothers. We recruited mothers who had a child aged 6 to 23 months and were available at home. When there was more than one child aged 6 to 23 months in a family, we included the youngest child. We excluded mothers who were not at home, were not willing to participate, or had a child younger than 6 months or older than 23 months.

### Study instrument

We followed the Pan American Health Organization (Regional Office for the Americas of the WHO) Process for the Promoting of Child Feeding (ProPAN) guideline for improving the diet and feeding practices of infants and young children between 6 and 23 months of age (21). In 2008, a WHO/UNICEF Expert Consultation recommended the ProPAN tool, a systematic guideline on developing specific infant feeding intervention, and stated that encouraging parents to feed their children with locally available and diverse nutritious products is an effective way to improve the nutritional quality of diets, and thus can reduce undernutrition in children (24). ProPAN has been implemented in several countries, but not in China. Therefore, we first translated the ProPAN guideline into Chinese and adapted it during three pilot studies to make it appropriate for the Chinese context.

### Interviewers and training

Two members of our study team (a nutritionist and senior nutritionist) translated and adapted the ProPAN guideline and acted as supervisors. We recruited six medical students for the 24-hour dietary recall survey. All interviewers had survey experience and the supervisors trained them for one day on ProPAN procedures for the study. The supervisors and local coordinators (village doctors and local teachers) conducted the market survey, food attribute exercise, and recipe creation exercise. Seven local coordinators carried out the first and second interview of the household trials of selected recipes. The supervisors spent one day training them to understand the questionnaires and to conduct the interviews.

### Sampling and sample size

Our calculated sample size was 98 children for the 24-hour dietary recall survey, based on core indicators of infant and young child feeding practices. We used an expected proportion for the minimum dietary diversity and the minimum meal frequency of 0.5 to conservatively estimate the sample size (5), as there were no previous data available for this county. We used a desired level of absolute precision of 0.14, an estimated design effect of 2, and an alpha error of 0.05. We used a random table in Excel to randomly selected five townships out of the nine townships in the county. Then we selected five villages in each selected township using the Probability Proportionate to Size sampling method. We excluded villages with the total population of fewer than 300, as these villages would not have provided enough children for our study. We selected all the children and their mothers who were available at home in the sampled villages as participants.

We used convenience sampling for the remaining parts of the study as recommended in the ProPAN guideline; a minimum number of five retailers for the market survey and 10 mothers of children aged 6 to 23 months for the other parts (21). For the market survey, we selected five different types of retail locations (two supermarkets, two canteens, and an open market) most frequently visited by mothers. We asked mothers which markets they usually went to and discussed with local coordinators which five markets to choose. For food attribute exercise, we interviewed 10 mothers with children aged 6-23 months. For the recipe creation exercise, we selected six to nine mothers for each age group (6 months, 7-8 months, 9-11 months, 12-17 months, and 18-23 months). For the household trial, we selected 6 to 7 mothers for each two recipes according to the different age groups.

### Procedures of recipe development

*24-hour dietary recall survey.* We carried out the 24-hour dietary recall survey from July 29 to July 31, 2011. The day before the survey, we asked mothers to put aside equal amounts of the same foods that their children ate that day. The interviewers used the 24-hour dietary recall ProPAN questionnaire to ask mothers about what the children ate and drank on the previous day.

*Market survey.* We discussed with the local coordinators which foods sold in the retail locations were of high nutritional value and could be consumed by children. We combined the foods that were selected in the discussions with the results of 24-hour dietary recall survey. We developed a food list that we could use for the market surveys. We interviewed retailers from the markets most frequently visited by mothers. We collected information on the food’s name, retail until, net weight, price, and seasonality (the month in which the food was available). We summarized the information about those food items.

*The key food list.* We developed a key food list, which included foods based on the results of 24-hour dietary recall survey and the market survey. We used the following criteria: 1) foods most frequently mentioned in the 24-hour dietary recall survey; 2) foods with a low frequency in the 24-hour dietary recall survey, but which are important nutrient sources and could potentially be used by mothers and consumed by children; 3) foods that have a high energy or nutrient value at low cost; 4) foods which could be grown or produced at home. We also listed the reasons why we chose the selected foods as the key foods and took a picture of each food product.

*Food attribute exercise.* We visited mothers with children aged 6-23 months in their homes from October 18 to October 19, 2011. One of the interviewers demonstrated a mother the pictures of the key foods and then interviewed her to obtain her perception on the food and her way of preparing it. The ProPAN food attribute interview guide was used and the interviews typically lasted 30 minutes. Interviews were digitally recorded, transcribed verbatim, and reviewed. Notes were taken during the interviews. The transcriptions were kept as Microsoft Word documents.

*Development of food combinations.* We compared the dietary values of all the food combinations to the Chinese dietary reference intake for children younger than two years. We chose the food combinations that could be used for a child aged 6-23 months for a whole day.

*Recipe creation exercise.* We spent two and a half days on five recipe creation exercises; each exercise took half a day. For each exercise, we selected mothers of one of the five age groups (6 months, 7-8 months, 9-11 months, 12-17 months, and 18-23 months), according to the different complementary feeding recommendations for each group. Each group included 6-9 mothers, with 2 or 3 mothers working together in subgroups.

Before each exercise, the senior nutritionist explained the purpose of the study and the feeding recommendations to mothers. Mothers were instructed to create three meals (breakfast, lunch, and dinner) according to the feeding recommendations and their normal cooking habits. We gave a one-day food combination to mothers and asked them to design three recipes for a whole day for their children. The supervisors reviewed each draft recipe designed by mothers to assess whether it was following the recommendations. Mothers cooked the dishes from the recipes that followed the recommendations. We also taught mothers how to mash meat with a blender. We asked mothers and their children to taste and comment on the dishes made by themselves and other mothers. The nutritionists assessed each recipe according to feeding recommendations, obtained children’s responses, and mothers’ perceptions, and identified the ten best recipes, which would be tested in the next stage.

*Household trials.* We evaluated the acceptability, compliance, and feasibility of the ten recommended recipes using ProPAN questionnaires. For every two recipes, 6-7 mothers were invited to test the recipes in their own homes for two weeks.

Before the household trial, we spent two days training mothers on how to test the recipes at home. In order to help the mothers understand the recipes better, we showed them videos explaining how to cook them at home. We sent cards to mothers with the following information: recipe pictures, information on why these recipes were healthy for their children, the ingredients, and cooking methods. We gave mothers a form and asked them to record the name of the recipe, which meal was fed to the child, and the amount of the food that the child ate, and to take a photograph of the foods they cooked. The form also served as a reminder.

We visited mothers three times after giving the recipes: at the first, sixth, and fourteenth day. During each visit, we asked mothers about their experiences with testing the recipes at home using a semi-structured ProPAN interview guide. To assess acceptability of the recipes, we asked mothers whether they were willing to continue using these recipes and whether they thought their children liked them. To assess compliance, we asked mothers about the number of times they used these recipes during the two weeks. To assess feasibility, we asked how they felt using these recipes, what did they liked or disliked about these recipes, whether they changed the recipes, what they changed, why they were or were not willing to continue using these recipes. When we found that mothers did not use recipes, we would explain the importance of the recipes and encourage them to continue using them. When mothers still did not want to use them, we gave up. We allowed mothers to use other recipes of their preference.

*Nutritional recipes.* We chose nutritional recipes for local children based on the opinions of mothers we collected during the household trials and advice from the nutritionists. We also developed a recipe booklet for local mothers to help them to improve their child feeding practices. In the booklet, we gave mothers suggestions for solutions to problems they might encounter in the household trials.

### Data analysis

We carried out the statistical analyses with SAS 9.1.0 for Windows (Statistical Analysis System Inc, Cary, NC, USA). We report proportions for the feeding practices. Data from the 24-hour dietary recall were entered into Microsoft Excel 2007 (Microsoft, Redmond, WA, USA). Frequencies of food consumption and the collected foods were counted manually. We used the Nutrisurvey software (25) and the Chinese food composition table (26) to calculate the nutritional values of food combinations for each age group. We manually analyzed the qualitative data and listed the most frequently mentioned perceptions of mothers.

The study was approved by the Ethical Committee of Capital Institute of Pediatrics in Beijing. We obtained oral and written informed consent from all the participants at each stage of our study.

## Results

### 24-hour dietary recall survey

A total of 110 mothers with children aged 6 to 23 months agreed to participate in the 24-hour recall survey. Five mothers could not participate because they did not know what their children ate on the previous day. The numbers of children in the two age groups (6-11 months and 12-23 months) were similar and the ratio of boys to girls was 1.5:1. The mean age of mothers was 29 (ranging from 20 to 46) and more than half of the mothers completed junior high school.

### Breastfeeding and complementary feeding

Most of the children (70.0%) were currently breastfed and only a minority of mothers prepared special meals for their children (22.7%). Although a majority of children (69.1%) were fed according to the minimum feeding frequency, dietary diversity (eating foods from at least four food groups a day) was quite poor as only one out of ten children (10.0%) was fed foods from at least four food groups. Very few currently breastfed (including mixed fed) children’s (3.9%) diet met both the recommendations for dietary frequency and diversity (this was caused by the fact that most children’s food did not meet the minimum dietary diversity).

### Foods consumed

A total of 31 food items were reported in the 24-hour dietary recall survey and 13 of them were consumed by 10.0% or more of the children (Table 1). The most popular food was eggs, which was given to over half of the children (50.0%). Other frequently eaten foods were grains, such as rice (34.5%), noodles (28.2%), maize flour (20.9%), and steamed bread (20.0%), and cookies (19.1%). Only a minority of children consumed meat (10.9%) and dairy products (21.8%) and very few children (1.8%) were given vitamin A-rich fruit and vegetables (mainly dark green leafy vegetables, carrots, and mangoes).

**Table 1:** Foods consumed by 10% or more of infants during the 24-h recall period

Food items	No. (%) of children (N = 110)	Number of times mentioned
Egg	55 (50.0)	73
Rice	38 (34.5)	53
Noodles	31 (28.2)	39
Maize flour	23 (20.9)	24
Steam bread	22 (20.0)	25
Cookies	21 (19.1)	25
Lactic acid drink	20 (18.2)	28
Cowpea angle (green beans)	20 (18.2)	21
Millet (type of grain)	18 (16.4)	24
Sausage	15 (13.6)	18
Infant formula	14 (12.7)	24
Cakes	14 (12.7)	14
Eggplant	12 (10.9)	12

### Market survey

We visited five markets and interviewed the retailers. Almost all the foods surveyed were available in the markets during the whole year, except sweet potato and Chinese cabbage which were only available in the autumn and winter. Grain, eggs, and vegetables were cheap, less 10 Yuan (1.59 USD) per Jin (equal to 500 g) and could be afforded by most families. Meats were more expensive than other foods, all of them were more than 10 Yuan per Jin, and mutton was the most expensive meat −25 Yuan (equal to 3.97 USD) per Jin.

### The key food list

We selected 27 foods for the key food list (Table 2). This list includes both foods that were frequently consumed by children and foods that were rarely eaten by children, but are of high nutritional value. We excluded the foods that children frequently ate but which had a low nutritional value, such as sausages, canned fish, sweet snacks, and the foods that were too hard for children to eat.

**Table 2:** The key food list and reasons for choosing foods

Frequently mentioned foods	Reasons for choosing foods		
	Mentioned in dietary recall survey	Available (in local markets)	Source of nutrients
Egg	Frequently	Yes	Proteins
Rice	Frequently	Yes	Carbohydrates
Noodles	Frequently	Yes	Carbohydrates
Maize flour	Frequently	Yes	Carbohydrates
Steam bread	Frequently	Yes	Carbohydrates
Cowpea angle(green bean)	Frequently	Summer and autumn	Vitamin A
Eggplant	Frequently	Yes	Vitamin B
Nutrient-rich foods not frequently mentioned by mothers			
Lean pork	Rarely	Yes	Iron
Chicken	Rarely	Yes	Iron
Pork liver	No	Yes	Iron and vitamin A
Tomato	Rarely	Yes	Vitamin A and C
Pork blood	No	Yes	Iron
Sweet potato	No	Yes, and also produced at home	Carbohydrates, proteins and calcium
Potato	Rarely	Yes	Carbohydrates
Tofu	Rarely	Yes	Proteins
Carrot	No	Yes	Vitamin A
Pumpkin	Rarely	Yes, and also produced at home	Carbohydrates, vitamin A
Rape	Rarely	Spring, summer, and autumn	Vitamin A and dietary fiber
Chinese cabbage	Rarely	Autumn and winter	Vitamin C and dietary fiber
Spinach	Rarely	Spring, summer and autumn	Vitamin A and dietary fiber
Pure milk	Rarely	Yes	Proteins and calcium
Yoghurt	Rarely	Yes	Proteins and calcium
Apple	Rarely	Yes	Vitamin C and dietary fiber
Banana	Rarely	Yes	Vitamin C and dietary fiber
Beans	Rarely	Yes	Proteins
Sesame paste	No	Yes	Calcium
Fat-rich food not mentioned above			
Oil	Frequently	Yes	Fat

### Food attribute exercise

We selected 10 key foods that children rarely ate, but which were of high nutritional value from three categories: meat (liver and lean meat), vegetables (pumpkin, rape, spinach, Chinese cabbage, sweet potato), and other foods (tofu, beans, and yoghurt). We interviewed 10 mothers (5 mothers with a child aged 6-11 months and 5 mothers with a child aged 12-23 months) on their reasons for not giving these foods. No mothers refused to participate in the interviews.

### Meat

The major reason mothers mentioned for not giving their children meat was that their children did not have teeth, and therefore mothers thought that their children could not chew and digest meat (Table 3). Most mothers said they would not feed their children meat until their children had teeth.

**Table 3:** Mothers’ perceptions of meat and feeding practices

Meat (No. of mothers asked)	No. of mothers who did not give the food to their child and reasons why	No. of mothers who gave the food to their child and reasons why	Reason why mothers did not give their child the food at six months	Conditions necessary to give the food to a child younger than two years of age
**Lean meat** **(10)**	**8, reasons:** 1. No teeth, cannot chew, and digest it (8/8)	**2, reasons:** 1. I want to improve the nutrition of my child (1/2) 2. The child can eat meat (1/2)	1. The child’s stomach was not good for lean meat (1/2)	1. When the child has teeth and can chew meat (7/8) 2. Do not know (1/8)
**Liver** **(10)**	**7, reasons:** 1. No teeth, cannot chew and digest it (4/7) 2. Do not know (1/10) 3. Too hard to swallow (1/7) 4. The child does not like to eat it (2/7) 5. I do not like to eat it, and do not prepare it for the child (1/7) 6. The child is allergic to liver (1/7)	**3, reasons:** 1. Liver can prevent anemia (2/3) 2. Liver is good for eyes (1/3)	1. Do not know (2/3) 2. The child was too young to digest liver before 9 mo and I feared choking (1/3)	1. When the child has teeth and can chew and digest it (5/7) 2. Do not know (2/7) 3. The child is able to eat (1/7)

### Vegetables

Mothers gave the same reason for not feeding their children vegetables: “the child cannot chew and digest it” (Table 4). Other reasons were that they only gave children foods they prepared for the whole family, that the vegetable was not in the season, or that the child did not like a particular vegetable.

**Table 4:** Mothers’ perceptions of vegetables and feeding practices

Vegetables (No. of mothers asked)	No. of mothers who did not give the food to their child and reasons why	No. of mothers who gave the food to their child and reasons why	Reason why mothers did not give their child the food at six months	Conditions necessary to feed the food to a child younger than two years of age
**Pumpkin** **(10)**	**2, reasons:** 1. It is not the season for pumpkin (1/2) 2. Pumpkin could not be used to make dishes at home (1/2)	**8, reasons:** 1. Do not know (1/8) 2. Learned from books (1/8) 3. It is soft and easy to digest (3/8) 4. When preparing it for family members, I also give it to the child (1/8) 5. Pumpkin is available at home (2/8)	1. Breast milk is enough till the child is nine months of age (1/8) 2. Pumpkin was available at home at that time (8 mo, 11 mo) (2/8) 3. The child did not like to eat it before one year (1/8) 4. Family members did not like to eat the food for children younger than one year (1/8) 5. Do not know (1/8)	1. Do not know (2/2)
**Rape** **(10)**	**7/10, reasons:** 1. The child cannot chew and digest it (5/7) 2. Do not have time to cook (1/7) 3. Family members don’t like to eat it (2/7) 4. Rape is rarely eaten at home, and I think that the child doesn’t like to eat rape (2/7) 5. Not produced at home (2/7) 6. It is not the season for rape (1/7)	**3/10, reasons:** 1. The child is able to eat it (1/3) 2. When preparing it for family members, I also give it to the child (1/3) 3. Should improve nutrition (1/3)	1. The child cannot chew rape before one year (1/3) 2. Breast milk is enough before the eight months of age (1/3) 3. The child did not like to eat it when he or she was six months old (1/3)	1. When the child has teeth and can chew rape (4/7) 2 Cooked for a longer time (2/7) 3. When rape is available at home (3/7) 3. Family members like to eat it (2/7)
**Spinach (10)**	**6/10, reasons:** 1. The child cannot chew and digest it (4/6) 2. Do not know (1/6) 3. The child does not like to eat (1/6)	**4/10, reasons:** 1. The child is able to eat (2/4) 2. When preparing it for family members, I also give it to the child (1/4) 3. Spinach was available at home (1/4) 4. Don’t know (1/4)	1. Too young to chew spinach before the age of one year (1/4) 2. Cannot eat spinach before the age of one year (1/4) 3. Breast milk is enough before the age of one year (1/4) 4. Breast milk is enough before the age of eight months (1/4)	1. When the child has teeth and can chew and digest spinach (6/6)
**Chinese cabbage** **(10)**	**0/10**	**10/10, reasons:** 1. Available at home (3/10) 2. Available at the market (1/10) 3. When preparing for family members, I also give it to the child (3/10) 4. Cheap (1/10) 5. Child has teeth and is able to eat (2/10) 6. Breast milk is not enough and the child should eat some foods (1/10)	1. No teeth and cannot chew and digest it (4/10) 2. Breast milk is enough before the age of 10 mo (1/10) 3. Should be given complementary foods at the age of seven or eight months (1/10)	
**Sweet potato** **(10)**	**6/10, reasons:** 1. It is not the season for sweet potato (3/6) 2. Child cannot digest sweet potato (1/6) 3. I do not like to eat it, and do not prepare it for the child (1/6) 4. Do not know (1/6)	**4/10, reasons:** The child can eat some foods (4/4)	1. Child cannot eat foods before the age of nine months (1/4) 2. It was not the season for sweet potato when the child was six months old (3/4)	1. The sweet potatoes are seasonally available (5/6) 2. Do not know (1/6)

### Other foods

All ten children were fed tofu; some mothers thought it was nutritious, one mother said “tofu is soft and the child can eat it,” and mothers said they could give it to their children when they prepared tofu for family members (Table 5). Most mothers thought children could not chew beans, because children did not have teeth. Most mothers did not give yoghurt to their children; the reasons for this were that some mothers thought breast milk was enough and some feared diarrhea.

**Table 5:** Mothers’ perceptions of other foods and feeding practices

Other foods (No. of mothers asked)	No. of mothers who did not give the foods to their child and reasons why	No. of mothers who gave the foods to their child and reasons why	Reason why mothers did not give their child the food at six months	Conditions necessary to feed the food to a child younger than two years of age
**Tofu** **(10)**	**0/10**	**10, reasons:** 1. Nutritious (2/10) 2. Tofu is soft and the child can eat it (3/10) 3. When preparing for family members, also gives it to the child (4/10) 4. Do not know (1/10)	1. Breast milk is enough for the child before eight months of age (2/10) 2. Breast milk is enough for the child before one year (1/10) 3. Child cannot eat at 6 mo of age (2/10) 4. Do not know (2/10)	
**Beans** **(10)**	**9, reasons:** 1. Do not know (1/9) 2. Do not know how to cook it (1/9) 3. The child did not like to eat it and spit it (1/9) 4. Not the season for bean (3/9) 5. No teeth and cannot chew (5/9) 6. Expensive (3/9)	**1, reason:** “I want my child to eat some vegetables” (1/1)	1. The child does not like to eat beans (1/11)	2. When the child has teeth and can chew and digest beans (5/9) 3. When beans are available (3/9) 4. When beans are cheap (1/9)
**Yoghurt** **(10)**	**9, reasons:** 1. Too thick and the mother fears that the child will choke (1/9) 2. Child did not like to eat it (2/9) 3. No need, breast milk is enough for the child (2/9) 4. Fear of diarrhea (2/9) 5. Do not know (3/9)	**1, reason:** Child should eat some foods (1/1)	1. Breastmilk is enough for the child before the age of one, and yoghurt is not nutritious (1/10)	2. When it is available at home (1/9) 3. Do not know (6/9) 4. Children like to eat it (2/9) 5. Breast milk is not enough (1/9) 6. When the child can digest foods well (1/9)

### Development of food combinations

According to the Chinese food composition table and the Chinese dietary reference intake for children younger than two years, we developed three food combinations for each of the five age groups (Supplementary files).

### Recipe creation exercise

Thirty-five mothers took part in the recipe creation exercise; all mothers who we approached were willing to participate. Mothers found it was easy to cook according to the feeding recommendations and that mashing meat with a blender was a good way to feed their children meat. However, as mothers used their normal cooking style, some recipes were only suitable for adults and not for children (not mashed enough), and we did not choose those recipes. We developed ten recipes for affordable, nutritional, and locally available dishes that can be used for complementary feeding of children aged 6 to 23 months. (Supplementary files).

### Household trials

Thirty-six mothers participated in the household trials; four mothers were not interviewed because they were busy or unwilling to participate. We report the acceptability, compliance, and feasibility of the ten tested recipes (Table 6). During the two weeks’ household trials, most mothers put the recipes into practice (around two times per week), and found these recipes nutritious, useful, and easy to cook. Mothers intended to continue using most of the recipes. Only 3 out of 6 mothers wanted to continue making one recipe, noodles with pork liver, eggplant, and potato. The other mothers said it was inconvenient to cook as many ingredients had to be used and children disliked it. Another reason given that may limit the use of the recipes was that some mothers said they did not have time to cook special foods for their children.

**Table 6:** Acceptability, compliance, and feasibility of the ten recipes tested

N	Recipes	Acceptability		Compliance		Feasibility		
		Number of mothers who intended to continue using it	Children’s responses	Number of mothers who put the recipe into practice	Average number of times that mothers used the recipe per week (2 weeks in total)	Compatibility with beliefs and knowledge	Cost in economic resources	Cost in time and effort
1	Porridge with pumpkin and rape^†^	6/6*	Liked eating it (6/6), could not eat when ill (1/6)	6/6	4	1. Nutritious (4/6) 2. Homestyle cooking, eating almost every day (3/6)	Inexpensive (5/6)	1. Easy to cook, takes little time and effort (6/6) 2. All the ingredients are available at home (3/6)
2	Rice porridge with tofu and rape^†^	7/7*	Liked (4/6), disliked (2/6)	6/6	3	1. Nutritious (6/7) 2. Homestyle cooking and no need to make special meals for children (1/7)	Inexpensive (7/7) Easy to buy (7/7)	1. Easy to cook, takes little time and effort (7/7)
3	Rice porridge with mashed pork liver and carrots	4/6	Liked (2/6), disliked due to the smell of pork liver (2/6)	4/6	1	1. Nutritious and good for children (4/6) 2. Children were too young to digest pork liver (3/6)	Raw pork liver is not easy to get from markets, but cooked liver can easily be bought (4/6)	1. Easy to cook (3/6) 2. Need a blender to make mashed liver (4/6)
4	Noodles with egg, tomato and rape^†^	6/6	Liked (5/6), disliked (1/6)	6/6	4	1. Nutritious (4/6) 2. When children liked it, mothers were willing to cook it	Inexpensive (6/6)	Simple and easy to cook, with little effort (6/6)
5	Noodles with pork liver, eggplant and potato	3/6	Liked (3/6), disliked (3/6)	6/6	2	1.Nutritious (6/6) 2. When children disliked it, mothers were not willing to cook it (3/6)	Pork liver is a bit expensive, but is acceptable (3/6)	1. Inconvenient to cook (3/6) 2. Need to prepare lots of ingredients (3/6)
6	Noodles with pork blood and carrots^†^	7/7	Child liked eating(6/7), disliked noodles (1/7)	6/7	2	1. Nutritious (7/7) 2. Child can eat pork blood easily (1/7) 3. Eating animal blood can prevent anemia (1/7)	1. Inexpensive (3/7) 2. Ingredients are easily bought from local markets (6/7)	1. Easy to cook. Take little time and effort (5/7) 2. Need to do special meal for child, inconvenient. (2/7)
7	Noodles with rape and agarics (fungus)	6/6	Liked (3/6), disliked (3/6)	6/6	2	1.Nutritious (6/6) 2. Eating rape or other vegetables can provide vitamins; eating agarics can provide iron(1/6)	1. Inexpensive (5/6)	Simple and easy to cook, with little effort (5/6)^‡^
8	Rice with chicken, eggplant, and potato	4/6	Liked (4/6), disliked eggplant and potato. (1/6), disliked rice (1/6)	6/6	2	1.Nutritious (5/6) 2.Home style cooking(1/6) 3. It is unnecessary to cook, because children disliked it (1/6) 3. Rice is dry, hard, and not easy to digest (3/6)	1. Inexpensive (5/6) 2. Ingredients are easily bought from local markets (1/6)	Simple (5/6)^‡^
9	Rice with lean pork and potato^†^	4/6	Liked (4/6), disliked rice (2/6)	6/6	1	1. Lots of ingredients and nutritious (6/6) 2. Children will be bored if they always eat the same food (1/6)	Pork is a bit expensive (2/6)	Need more efforts to prepare lots ingredients (4/6)
10	Rice enriched with tomato and pork liver^†^	4/6	Liked (4/6), disliked rice (2/6)	6/6	2	1. Nutritious (6/6) 2. Color is good (1/6)	1. Inexpensive (3/6) 2. Raw pork liver is not easy to get from markets (2/6)	Simple and easy to cook, with little efforts (5/6)

*Some of recipes were tested by six mothers, and some were tested by seven mothers.

†Six recipes we finally selected.

‡One of the mothers did not mention on the “Cost in time and effort” topic.

### Nutritional recipes

We selected six recipes that had good acceptability, feasibility, and compliance. Some of the ten recipes could be cooked by the same method, but with different ingredients. Therefore, we combined ingredients from different recipes into one recipe. For example the preparation methods for the recipe “rice with chicken, eggplant, and potato” was similar to the recipe “rice with lean pork and potato,” so we combined the ingredients into one recipe “rice with lean pork and potato.” We created a recipe booklet in which the quantity of ingredients and the preparation method for each recipe was described (Supplementary files). The booklet also includes suggestions on how to solve problems which mothers could encounter (based on problems mothers encountered during the household trials). For example, we recommended mothers to add mashed meat to some recipes to increase the iron content. Each recipe in the booklet is recommended as one meal for one child and recipe combinations are recommended for each age group. An example of a daily menu is rice porridge with tofu and rape for breakfast, rice with tomato and pork liver for lunch, and noodles with egg, tomato, and rape for supper. This recipe combination meets most of the daily recommended nutrients for a child aged 6-23 months a day (Table 7). We recommended mothers to give the child healthy snacks, such as yoghurt or fruit, in between the meals to fill the nutritional gaps.

**Table 7:** Nutritional value of example one-day recipe combination for children aged 12-23 mo*

Nutrient	Nutritional value	Recommended daily intake (China)	Percentage of daily needs
Energy (kcal)	932.6	1050	88.8
Protein (g)	36.2	35	103.4
Fat (g)	26.3	33.3-44.4	79.0
Vitamin A (μg)	2658.2^†^	500	531.6
Vitamin B1 (mg)	0.4	0.6	66.7
Vitamin B2 (mg)	1.34	0.6	223.3
Calcium (mg)	56.3	400	114.1
Iron (mg)	11.4	12	95.0
Zinc (mg)	5.5	9	61.1

*Compared with the recommended daily intake (China), the nutritional value of the recipe combination cannot reach 100% of recommendation. However, mothers were recommended to add fruits, yoghurt, or healthy snacks between two meals to compensate for the gap.

^†^We used pork liver as an ingredient, which contains high vitamin A content (4972 μg/100g).

## Discussion

Suboptimal infant feeding practices are an important, but unexplored issue in China. We identified locally available, nutritious, and affordable foods and developed six recipes with the potential to meet most of the nutritional need of children aged 6-23 months in Wuyi County in China. Most of recipes were acceptable, feasible, and mothers complied with them during the two-week study period.

An important focus of the study was to increase the iron consumption by encouraging mothers to feed their children iron-rich foods such as meat. Evidence shows that giving meat to children plays a paramount role in treating anemia and undernutrition, as meat is the first and most effective choice (27). Furthermore, eating meat is related to a greater nutrient intake and higher dietary quality (28). Eating vegetables is also important because they contain vitamin C, known to improve iron absorption (29). However, only a minority of children was given meat and vegetables; a possible reason for this was that most mothers thought young children cannot eat meat and vegetables, because they do not have teeth and cannot digest these foods. When mothers saw how to prepare mashed foods and how well their children accepted it, most of them were keen on cooking mashed meat and vegetables for their children.

Between 1985 and 2011, Chinese children’s average height considerably increased compared to other developing countries (30), but China is still home to the second largest population of stunted children globally (31). Improving the diversity of children’s diet increases their average height-for-age z-score (32-34). The use of locally produced nutritious foods can increase dietary diversity in any setting (24,35). A cluster-randomized trial in China using a booklet with recipes based on local foods showed improved dietary diversity (36). However, detailed development of the recipes was not given. A study in Mali showed that using local foods was more likely to be acceptable, affordable, feasible, and sustainable, because these foods were culturally appropriate and consumed on a daily basis (37). Preparing the recommended recipes should require little time and be affordable, so that mothers are willing to use the recipes during and after the study. A study in Bangladesh found that caregivers were more likely to adopt messages that promoted enriched foods requiring little time and money and were likely to ignore messages that promoted foods that were expensive and required more preparation time (38). In both the 24-recall survey and household trials, we found that mothers rarely cooked special foods for their children. To solve this problem, most of recipes were improved by using home style cooking methods; mothers reported this was convenient to do. Our nutritionists improved these recipes and mothers found it very easy to cook them at home. Some mothers had incorrect knowledge on infant feeding; for example they stopped using the recipes when their children got ill during the household trials. We tried to explain to continue feeding children during the illness and gave solutions for other problems in our recipe booklet.

Our study has some limitations and strengths. Our recipe booklet was based on local foods and dietary practices only in Wuyi County and may only be used in settings that have similar food resources and dietary habits. However, people in other settings in China can use this to develop their own recipes for infants and young children. While we developed only six recipes, most of the ingredients can be replaced by other foods from the same food group to make more recipes with similar nutritional values. This would overcome a barrier to using the recipes that mothers mentioned: children become bored of the foods. Another limitation of the recipes is that the nutritional value of one-day recipe combinations is based on three meals per day. In fact, mothers could not use the recipes for three meals a day every day. This gap can be filled by adding in-home complementary food fortification (such as micronutrient powders). Evidence showed that home fortification of foods with multiple micronutrient powders is an effective intervention to reduce anemia, iron, and vitamin A deficiency in young children (35,39). This is a formative study, which could not assess whether the nutritional status of children was improved by using the recipe booklet as a source of complementary feeding advice. A strength of this study is that both quantitative and qualitative data were collected to assess the local foods and to gain a better understanding of the perception of mothers on key foods and recipes. Thereby, we could give mothers personal advice and support. Our study described a step-by-step process of how to develop local recipes, which could help others in developing their interventions to improve the nutritional status of children.

Future studies need to be conducted in areas with varying food availability. Local food productivity patterns such as grain output can be used as a rough proxy to identify areas of similar food availability, which in turn can guide future studies (40). Also studies need to assess the compliance of mothers with the recipes for a longer period of time and the effectiveness of these recipes in improving the nutritional status of targeted children. In addition, this methodology can be used to address obesity problems. While China was among the 30% of countries with the lowest BMIs in 2008, BMIs are increasing (41). With growing wealth, obesity may be a future problem in which promoting healthy child nutrition can offer a key solution (42).

This is the first study in China using a systematic evidence-based approach to develop complementary feeding recipes that have the potential to improve the nutritional status of children. Our study gave insight into how nutritional recipes could be created and tailored to a local context and showed the barriers mothers faced in feeding their children with nutritious food. We gave mothers correct feeding knowledge and supported them in cooking appropriate nutritional foods for their children. The recipes were acceptable, feasible, and mothers used them during our short study period. This needs to be studied further so that long-term effects on the nutritional status of children can be assessed.
